# Temporary Insanity, from the Use of Bromide of Potassium

**Published:** 1870-06

**Authors:** F. K. Bailey

**Affiliations:** Knoxville, Tenn.


### TEMPORARY INSANITY, FROM THE USE OF BROMIDE OF POTASSIUM.

KNOXVILLE, TENN., *May, 1870.*

TO THE EDITOR OF THE MEDICAL EXAMINER:

In Prof. W. A. Hammond's *Psychological Journal*, for Jan., 1869, is an article from the pen of the editor "on Some of the Effects of Bromide of Potassium, in Large Doses." It is but lately that I met with the article in question; and on reading it, it seemed proper to add a few observations, suggested since writing my remarks upon the bromides, in the January number of the EXAMINER. I then alluded to the case of a colored man, laboring under epilepsy, with some indications of mental aberration.

From Dr. D. T. Boynton, of this city, who had the case in charge previous to my coming here, I learn that the man had taken, during the summer of 1867, bromide potassium, in doses of twenty grains, two or three times daily, quite regularly, and that it was while taking the salt thus freely, that the peculiar form of insanity first manifested itself. Dr. B., at the time, attributed these symptoms to the epilepsy, together with excitement, growing out of local politics affecting his race, and to religious enthusiasm.

He was taking the bromide when I first saw him, Oct. 1st, 1867, and at that time his insanity was most obvious. I only gave eight or ten grains at a dose, after assuming the care of his case; and it was continued for a month or two, but, perhaps, not unremittingly.

I will add, however, that this man appears to be slowly recovering his health. The epileptic phenomena are mainly slight attacks, which he can anticipate, and generally is able to reach his home before becoming insensible.

The fits recur only about once in two months, and his mental faculties are less impaired. Much good has been accomplished, in this case, by carefully watching the condition of the alimentary canal and the timely use of laxatives.

A second case bearing upon this subject has recently come to my notice. A few months ago, a family removed to this city from Cincinnati. One of the number, a young man, aged 17, has had epilepsy five years. I am informed by Mr. A., an older brother, who is an intelligent druggist, that he had scarlatina ten years ago, from which he slowly recovered. Soon after convalescence, he began to complain of feverishness, attended with a flushed countenance. These attacks were only occasional at first, and lasting but a short time. He was treated, by different medical men, with antiperiodics and tonics, but with no permanent effect.

Finally, these symptoms gradually merged into confirmed epilepsy. Soon after coming here, Mr. A. put up, as he informs me, the following combination, upon his own responsibility:—

℞. Potassii Bromidi, }<br>

Ammonii " } āā, ------------------ ʒj.<br>

Fluid Ext. Valerian, -------------------- ℥iij.<br>

Aquas Puræ, ------------------------------ ℥xiij.<br>

Strychniar Sul.,------------------------- gr. j.

M. Sig. Tablespoonful three times daily. Very soon after its use was commenced, the fits became less frequent, so that instead of five or more in a day, he had but one in three weeks.

In less than a month after, however, he began to show signs of mental excitement; and, one day, a servant ran hurredly to announce to Mr. A., that "*Will* had gone crazy."

Dr. Boynton, whose office stood adjoining the house, was requested to see him, and at once decided that he was laboring under the effects of the bromide.

He was very much excited, and drove his mother and sister from the house. Complained of an unpleasant sensation in the forehead, extending towards the left orbit. He was inclined to run about, and there were also involuntary and convulsive movements of the lower limbs. He would run about in a circle and kick at both persons and things.

This continued for some hours; but the exhibition of a full dose of powdered opium soon produced quiet.

Since that time (which was a month or more ago), he has been more free from fits than for a long time.

I have suggested, in this case, a continuance of the bromide, in doses of eight or ten grains, three times daily. The question might arise whether the convulsive movements of the limbs and disposition to kick and run about were not the effects of strychnine. The dose was but $\frac{1}{32}$ part of a grain, and cannot be considered excessive. F. K. BAILEY.

